# Supplementary material for: Molecular and anatomical organization of the dorsal raphe nucleus
Source: eLife. 2019 Aug 14;8:e46464. doi: 10.7554/eLife.46464 (PMC6726424; doi:10.7554/eLife.46464)
Supplement: Supplementary file 1. — Several examples of genes used to identify each of the major cell types/classes are listed. Slc17a7 (VGluT1) is typically used to identify glutamatergic neurons but was not detected in our scRNA-seq dataset, and was therefore excluded from this list. [file elife-46464-supp1.docx]

**Table S1**

Genes for identification of major cell types

| **Cell class/type** | **Identifying genes** |
| --- | --- |
| Neurons | *Snap25*, *Syn1*, *Rbfox3* |
| Peptidergic neurons | *Ucn*, *Cartpt*, *Postn* |
| Glutamatergic neurons | *Slc17a6*, *Slc17a8* |
| GABAergic neurons | *Slc32a1*, *Slc6a1*, *Gad1*, *Gad2* |
| Dopaminergic neurons | *Slc6a3*, *Th*, *Slc18a2* |
| Serotonergic neurons | *Slc6a4*, *Tph2*, *Fev*, *Slc18a2* |
| Mature oligodendrocytes | *Olig1*, *Mbp*, *Mog*, *Opalin* |
| Differentiating oligodendrocytes | *Olig1*, *Gpr17^high^*, *Enpp6* |
| Polydendrocytes (OPCs) | *Olig1*, *Pdgfra*, *Cspg5*, *C1ql1*, *Gpr17^med^* |
| Astrocytes | *Slc6a11*, *Slc6a9*, *Aqp4*, *Gja1* |
| Ependymal cells | *Hdc*, *Cdhr4*, *Ucma* |
| Lymphocytes | *Cd52*, *Trbc2*, *Nkg7* |
| Microglia | *Cx3cr1*, *Tmem119*, *P2ry12* |
| Macrophages | *Mrc1*, *Ms4a7* |
| Fibroblasts/mesenchymal cells | *Col3a1*, *Dcn*, *Pdgfra*, *Pdgfrb* |
| Endothelial cells | *Cldn5*, *Flt1*, *Pecam1* |
| Pericytes | *Pdgfrb, Abcc9*, *Kcnj8* |
| Smooth muscle cells | *Tagln*, *Acta2*, *Myh11* |
